# Confronting microaggressions in the medical field for a more inclusive future

**DOI:** 10.1016/j.jvsv.2024.101818

**Published:** 2024-06-18

**Authors:** Omar Abdel Kerim, Anil Hingorani

**Affiliations:** a University of Miami Miller School of Medicine, Miami, FL; b Vascular Institute of New York, Brooklyn, NY

No less than a day after being asked to write this article on microaggressions, a patient joyfully told my attending, a Jamaican man of Chinese ancestry, “My primary doctor is Japanese too, you guys make great doctors!” The attending expertly redirected the situation to begin discussing the patient’s discharge plan, while the team and I managed our discomfort, making it very clear to us that this was not his first and will not be his last time experiencing such a situation.

In an attempt to anecdotally establish the prevalence of microaggressions in the medical field, I asked my peers and superiors, who comprise medical students, residents, and attendings, if they’ve ever experienced microaggressions in the workplace. With little elaboration, each immediately began exhaling frustrations at me, discussing numerous scenarios where they have been victims of microaggressions. Some common scenarios to note: “where are you really from?” “I trust your kind,” “I think it’s great that you want to pursue trauma surgery, but when do you plan to have kids?” or speaking to the male intern instead of the female attending.

Dr Anil Hingorani and Dr LeAnn Chaves recently presented contrasting viewpoints on the subject of microaggressions and their impact at the 2023 Society for Clinical Vascular Surgery meeting. Microaggressions, as defined by Sue et al,^1^ are subtle, often unintentional comments with underlying demeaning and insulting undertones, typically made by individuals who may not be aware of their effects. These are often related to their identity such as race, ethnicity, socioeconomic status, or sexual orientation. An example of such a microaggression can be found in the first sentence of this paragraph, where I list Dr Hingorani, a male surgeon, before Dr Chaves, a female surgeon. This ordering unintentionally prioritizes Dr Hingorani’s name, representing a form of sexist microaggression. This example highlights that microaggressions can manifest in various forms and directions, even from a medical student, like myself, towards a distinguished vascular surgeon like Dr Chaves. These incidents can occur bidirectionally between patients and physicians, trainees and supervisors, and peer to peer.

The ubiquity of microaggressions can possibly be dismissed if it were not for the impact it has on representation, burnout, turnover, and overall well-being of underrepresented groups in surgical fields. Numerous studies have documented the association between workplace microaggressions and these factors.^2^^,^^3^^,^^4^^,^^5^

In light of this pervasive issue, strategies have been developed to effectively address microaggressions when encountered during one’s professional journey. One such approach is the “Stop, Talk, Roll” framework.^6^ This approach is particularly valuable for medical students and residents when confronting microaggressions from team members or patients. The “Stop, Talk, Roll” framework advises individuals to defuse the immediate situation by either temporarily setting it aside or creating an exit strategy to allow for introspection and assessment. This approach recognizes that microaggressive comments can trigger intense emotional reactions and encourages the targeted individuals to distance themselves to return to the matter with a clearer perspective. They are urged to take time to process the incident to determine whether it necessitates a response.

This introspective process is especially crucial when addressing microaggressions committed by superiors, as these situations require delicacy and thoughtful handling. If, after a period of reflection, the individual finds themselves untroubled by the comments, they can continue with their daily activities. However, if they conclude that the situation demands further action, they can proceed with the “Talk” phase. This “pick your battles” approach is especially apt in a health care setting, where peer perception and feedback are sacrosanct.

When dealing with a microaggression from a supervising physician that warrants action, the individual should “call in” the physician, in a private and confidential setting.^7^ During this conversation, individuals should respect the hierarchical structure while candidly expressing their concerns. The tone of conversation should be nonpunitive with the goal of mutual understanding and respect.

The final step in the “Stop, Talk, Roll” framework is to “Roll”. In the context of patient interactions, “Rolling” entails scheduling an appointment with a counselor or utilizing peer-support resources provided by the institution to address any lingering emotional effects of the microaggression. When dealing with a microaggression involving a physician, individuals should again engage in introspection. If the situation remains unresolved, it may be appropriate to contact the previously mentioned resources for further guidance and steps forward.

By adopting such proactive approaches, health care professionals can effectively address and mitigate the impact of microaggressions in their careers, promoting a more inclusive and psychologically safe environment for all involved.

To avoid microaggression and the escalation of the situations, superiors and employers should aim to include microaffirmations in the workplace, by providing positive feedback when possible.^8^ More importantly, those who find themselves committing a microaggression should adopt an “ASSIST” approach rather than a defensive one. The acronym stands for **A**cknowledging possible bias, **S**eeking feedback, saying **S**orry, appreciating **I**mpact over intent, and **T**hank them for respectfully bringing it up.

Our reactions to having committed or experiencing a microaggression have major impacts on its outcome; it does not need to be an entirely negative scenario for both parties. How we approach the situation as perpetrators or victims determines whether it is a conflict or an opportunity for growth and learning.

## Disclosures

None.

## References

[1] Sue D.W., Capodilupo C.M., Torino G.C. (2007). Racial microaggressions in everyday life: implications for clinical practice. Am Psychol.

[2] Alimi Y., Bevilacqua L.A., Snyder R.A. (2023). The elephant in the room: racial microaggressions and implicit bias in surgical training. Ann surg.

[3] Carino Mason M.R., Pandya S., Joshi P., Cai N., Murdock C.J., Hui-Chou H.G. (2023). Perceptions of racial and gender microaggressions in an academic orthopaedic department. JB JS Open Access.

[4] MacIntosh T., Hernandez M., Mehta A.S. (2022). Identifying, addressing, and eliminating microaggressions in Healthcare. HCA Healthc J Med.

[5] Neha T., Sudol M. (2021). Prevalence and nature of sexist and racial/ethnic microaggressions. JAMA Surg.

[6] Cheng S.M. (2017). Stop, Talk, Roll: how to deal with tough communication exchanges in the medical workplace. Georgetown university school of medicine. https://som.georgetown.edu/diversityequityandinclusion/odi-initiatives/stoptalkroll/.

[7] Woods F.A., Ruscher J.B. (2021). 'calling-out' vs. 'calling-in' prejudice: confrontation style affects inferred motive and expected outcomes. Br J Soc Psychol.

[8] Stillman J. (2021). 5 tiny but impactful “microaffirmations” to make everyone on your team feel valued and included. Inc. https://www.inc.com/jessica-stillman/inclusion-microaffirmations-melinda-briana-epler.html.

